# Vertebral Fragility in Monoclonal Gammopathy of Undetermined Significance Despite Denosumab: Lessons From a Case

**DOI:** 10.7759/cureus.117844

**Published:** 2026-10-13

**Authors:** Evangelia Papakitsou, Alexandos Gkiokas, Christos Zafeiris, Emmanouel Tsafantakis, Marie-Christine Kyrtsonis

**Affiliations:** 1 Department of Trauma and Orthopaedics, Our Lady of Lourdes Hospital, Drogheda, IRL; 2 Hematology Section, First Department of Propaedeutic Internal Medicine, Laiko General Hospital, National Kapodistrian University of Athens, Athens, GRC; 3 Department of Orthopaedics and Spine Surgery, Metropolitan General Hospital, Athens, GRC

**Keywords:** balloon kyphoplasty, denosumab, monoclonal gammopathy of undetermined significance (mgus), osteoporosis therapy, osteoporotic vertebral fractures, prophylactic kyphoplasty, rebound phenomenon, smoldering myeloma, vertebral osteonecrosis

## Abstract

Evidence on antiosteoporotic therapies in patients with precursor stages of myeloma remains rare. Denosumab (DMAb) use in this setting is extrapolated from postmenopausal osteoporosis trials. We present an 81-year-old woman with known high-risk IgG/κ monoclonal gammopathy of undetermined significance (MGUS) who transitioned to low-risk smoldering multiple myeloma (SMM) and developed multiple vertebral fractures while receiving DMAb 60 mg.

The patient presented two months after an L4 spontaneous fracture, with severe low back pain impairing mobilization. Ten months earlier, she had sustained an L3 refracture. The fractures occurred at six months post-DMAb injections that were sequentially delayed by approximately seven to 10 weeks, respectively. Her disease had been reclassified as low-risk SMM 18 months earlier (bone marrow plasma cells 15%). At presentation, M-protein (1.93 g/dL) and free light chain (FLC) ratio (5.11) were rising but below the 20/2/20 thresholds. A new lumbar spine CT revealed L1 collapse with vacuum cleft and showed progression of the L4 fracture and the L3 refracture. DMAb was already 10 weeks delayed. S-NTx (13.5 nmBCE) and S-PINP (40.4 ng/mL) were elevated above the median premenopausal values (12.6 nmBCE and 37.5 ng/mL, respectively), consistent with the rebound phenomenon.

Given the prolonged DMAb exposure (four years), the delayed timing of the switch (8.5 months after the last dose), and the very high imminent fracture risk, resumption of DMAb was considered preferable to transition to zoledronate. Multilevel percutaneous balloon kyphoplasty was performed at L1, L4, and L3 refracture to reduce pain and preserve or gain height where possible. Intraoperative biopsies were received from L1, L2, and iliac bone. Adjacent fractures with cement, along with poor bone quality, indicated prophylactic cementation of L2. Biopsies confirmed L1 osteonecrosis and showed no evidence of myeloma. Despite the fracture burden and lack of a whole-body MRI, the patient remained under surveillance, since she was classified as having low-to-intermediate risk SMM.

Administering DMAb at approximately six months + 6.9 weeks of DMAb delay did not prevent the L3 refracture six months later. Thus, DMAb was given at shortened intervals (three months, then every four months) for one year and was monitored with S-NTx and PINP. This regimen attenuated bone resorption (S-NTx (7.5 to 8.4 nmBCE)) but did not suppress it below the lower premenopausal level before the next injection. PINP decreased below the median premenopausal value after one year of therapy. Post-kyphoplasty, a new L2 superior end plate fracture occurred within six months. At nine-month follow-up, a new bone marrow biopsy showed 10% bone marrow clonal plasma cells (BMPCs), and whole-body low-dose CT was negative for lytic lesions. L1 retropulsion progressed without neurologic symptoms. After three years on DMAb 60 mg every six months, no new fractures or neurologic complications occurred.

This case highlights the clinical ambiguity when increasing disease burden in patients with MGUS/SMM overlaps with the DMAb-related rebound phenomenon. Additionally, it underscores the gap in evidence-based treatment strategies for osteoporosis in SMM. Percutaneous kyphoplasty combined with monitored denosumab may be a viable option for patients with MGUS/SMM, very high imminent fracture risk and no neurologic deficits, if intervention is indicated.

## Introduction

Myeloma precursors are mainly asymptomatic, and their management depends on disease burden and the risk of progression to active multiple myeloma (MM). Monoclonal gammopathy of undetermined significance (MGUS) is characterized by the presence of serum M-protein <3 g/dL, bone marrow clonal plasma cells (BMPCs) <10%, absence of myeloma end-organ damage (CRAB), and absence of B-cell lymphoma or other M-protein-producing disease. It affects 5.3% of adults over 70 years of age, and it carries a 1% annual risk for progression to MM [1]. Smoldering multiple myeloma (SMM), with M-protein ≥3 g/dL and BMPCs 10-60%, progresses at approximately 10% during the first five years. Hematologic management is surveillance-based for low- and intermediate-risk SMM [1,2]. High-risk SMM patients likely harbor an evolving malignant clone. Crossing SLiM thresholds (BMPC ≥60%, a serum free light chain (FLC) ratio ≥100, or at least one focal MRI lesion) reclassifies MGUS/SMM as MM requiring systemic therapy.

Osteoporosis is increasingly recognized as a clinical component of myeloma precursor states. MGUS confers a 2.7-fold increased five-year hazard ratio for vertebral/pelvic fractures [3]. Conversely, ~15% of patients with acute osteoporotic vertebral fractures (OVFxs) referred to endocrinology were found to have MGUS [4]. Bone fragility is related to diffuse micro-architectural deterioration (bone expansion and cortical/trabecular thinning) that arises from increased osteoclast activity and suppressed bone formation mediated by clonal plasma cells [5]. Within patients with MGUS, fracture risk is variable, and selected cases may manifest "acute osteoporosis" and significant skeletal disease [6]. Evidence regarding the effectiveness of antiosteoporotic agents in patients with MGUS is limited. Based on two small RCTs [7,8], the International Myeloma Working Group (IMWG) recommends alendronate 70 mg PO weekly or zoledronic acid (ZOL) 4 mg IV every six months, and standard osteoporosis monitoring (grade C) [2].

An osteoporotic-appearing vertebral fracture in patients with SMM is often the first sign of disease progression, with over 50% of such patients progressing to MM within 18 months [9]. High-risk SMM patients are now candidates for early intervention with daratumumab (National Comprehensive Cancer Network Clinical Practice Guidelines (NCCN), 2026) or lenalidomide [10], reflecting a shift from the historical standard of surveillance or enrollment in a clinical trial [10]. Fracture management therefore requires distinguishing patients who need systemic hematologic therapy from those who need bone-modifying treatment alone. Due to the rapid transition to MM and the small numbers of patients with SMM, there is a lack of evidence on osteoporosis management in patients with SMM who do not require systemic therapy. Current consensus favors ZOL IV yearly [10] or every six months, or ideally in the context of a clinical trial [2]. Although recommended by the IMWG (grade D, panel consensus) [2], denosumab (DMAb) use in patients with MGUS or SMM not requiring systemic therapy is off-label, extrapolated from postmenopausal osteoporosis. Furthermore, strategies to exit DMAb might not apply to this setting.

We report a case of a woman with osteoporosis and high-risk MGUS (BMPCs 10%) who was switched to DMAb 60 mg after fracturing on alendronate. She subsequently progressed to low-risk SMM, and within a year developed a cascade of vertebral fractures. History of brief delays in DMAb dosing and the absence of whole-body MRI clouded the decision on systemic therapy. Additionally, resuming DMAb 60 mg after the initial delay did not prevent OVFxs and raised concerns about the effectiveness of the postmenopausal scheme. The case highlights the clinical ambiguity in managing patients with MGUS/SMM with DMAb, since disease progression may overlap with the rebound phenomenon.

For acute/subacute OVFxs without instability, the North American Spine Society (NASS) guidelines support cement augmentation when pain is unmanageable or compromises independent mobilization for three to six weeks [11]. The DGOU-AO tool (Working Group for Osteoporotic Fractures of the Spine Section of the German Society for Orthopaedics and Trauma) offers a validated score-based approach to select patients for operative treatment. It is based on fracture morphology, pain, mobility, deformity progression, and frailty. Severe osteoporosis suggests a lower likelihood to benefit from conservative treatment [12]. Rebound fractures were traditionally considered a relative contraindication to vertebral augmentation. Our case is also a paradigm of management of OVFxs in elderly patients with rebound OVFxs who do not have neurologic deficits.

## Case presentation

An 81-year-old woman with a history of IgG/κ MGUS and osteoporosis presented with severe back pain that impaired independent ambulation, two months after sustaining a spontaneous L4 fracture. Her clinical fracture history included an L3 fracture seven years ago, a shoulder fracture four years ago, and a refracture at L3 10 months ago. Following the shoulder fracture, the patient was switched from alendronate to DMAb. The last two injections of DMAb were administered 16 and eight months before presentation, having been delayed by approximately seven and 10 weeks, respectively, due to the pandemic lockdown (Figure 1). Her last bone marrow biopsy, performed 18 months earlier, formally reclassified her into the SMM category. It showed 15% mature plasma cells (CD20-, CD56-), with clonal IgG/κ expression and 15% bone marrow infiltration by small B lymphocytes (CD20+, CD3-) clonal for κ light chain, up from 10% in BMPCs 2.5 years earlier. Her medical history was notable for chronic kidney disease (CKD) stage 3b and longstanding mixed urinary incontinence. She resided with family and was an independent community ambulator at baseline. An old elbow implant precluded the use of MRI. 

**Figure 1:**
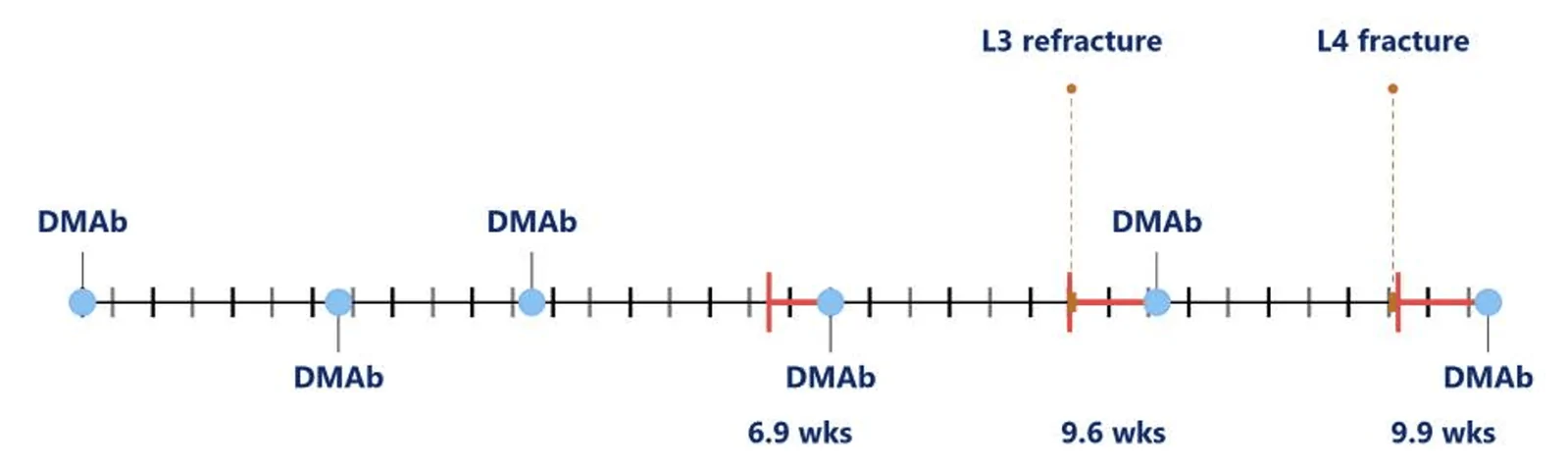
Timeline of denosumab administration and fractures. L3 refracture and L4 fracture appeared at approximately 10 and two months prior to presentation, respectively. Both fractures occurred consecutively at six months after denosumab (DMAb) injections. The injections were approximately seven and 10 weeks (wks) delayed. At presentation, DMAb was again 2.5 months (9.9 weeks) delayed. Ticks represent one month; delays are flagged in red.

Physical examination showed thoracolumbar kyphosis and diffuse tenderness at the lower thoracic and lumbar spine, without neurologic deficits. Review of available imaging showed a new 40% biconcave compression at L3, 10 months prior (Figures 2A-2B) that did not exist on whole-body low-dose CT (WB LDCT) eight months earlier (Figure 2C) and the primary L3 fracture (Figure 2D).

**Figure 2:**
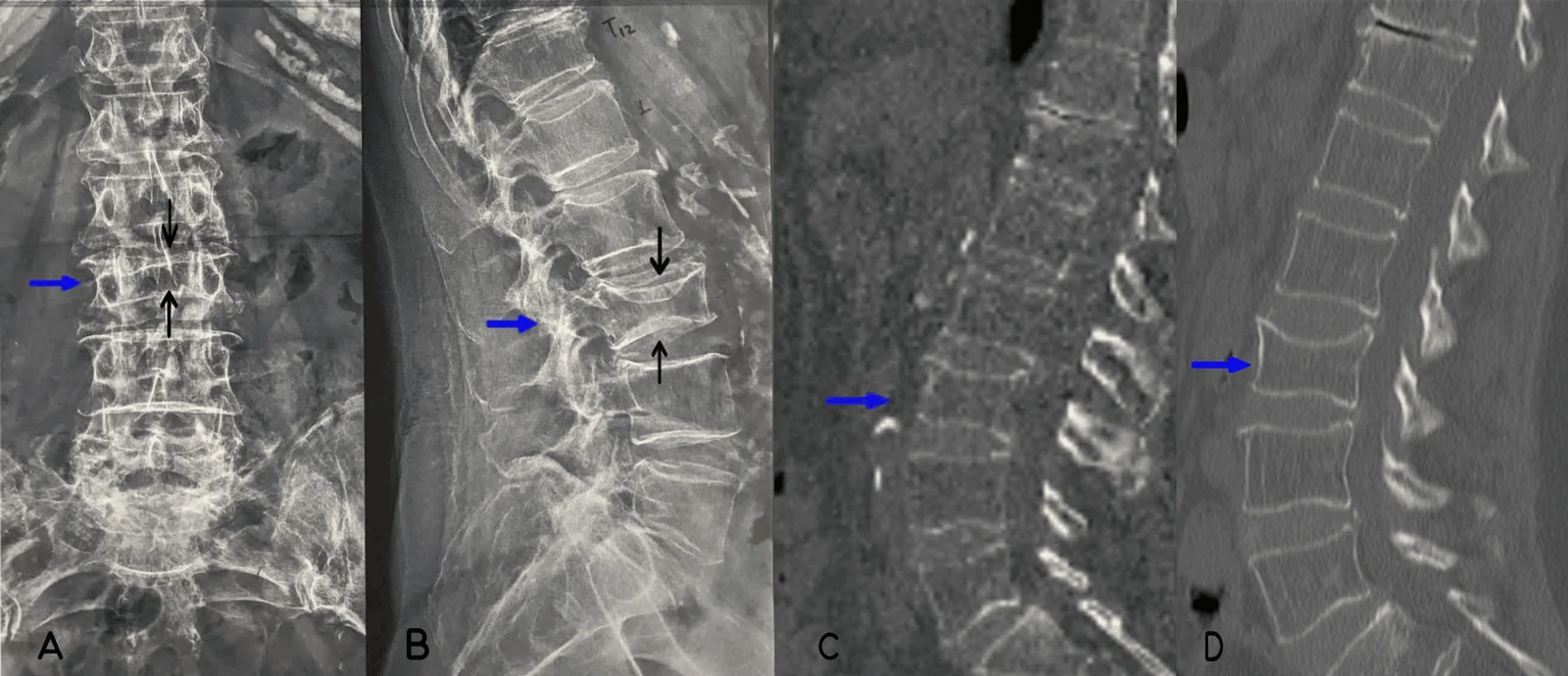
Natural course of L3 fracture over time. (A,B) L3 (blue arrow) refracture (black arrows) on lumbar spine X-rays (anteroposterior, lateral), 10 months prior to presentation. (C) L3 deformity with anterior and posterior wall height loss and superior end-plate compression, on WB LDCT, eight months earlier. (D) Superior end-plate fracture with significant preservation of anterior and posterior wall seven years ago.

A new lumbar spine CT scan (Figure 3A) revealed collapse of L1 with a vacuum cleft and mild retropulsion into the spinal canal, progression of the subacute L4 fracture first depicted on CT 2 months earlier (Figure 3B), and progression of the L3 refracture compared to X-rays 10 months earlier (Figures 2A-2B). No lytic lesions were visible. The fractures were classified as osteoporotic fractures (OF) type 4, using the DGOU/AO classification [12]. 

**Figure 3:**
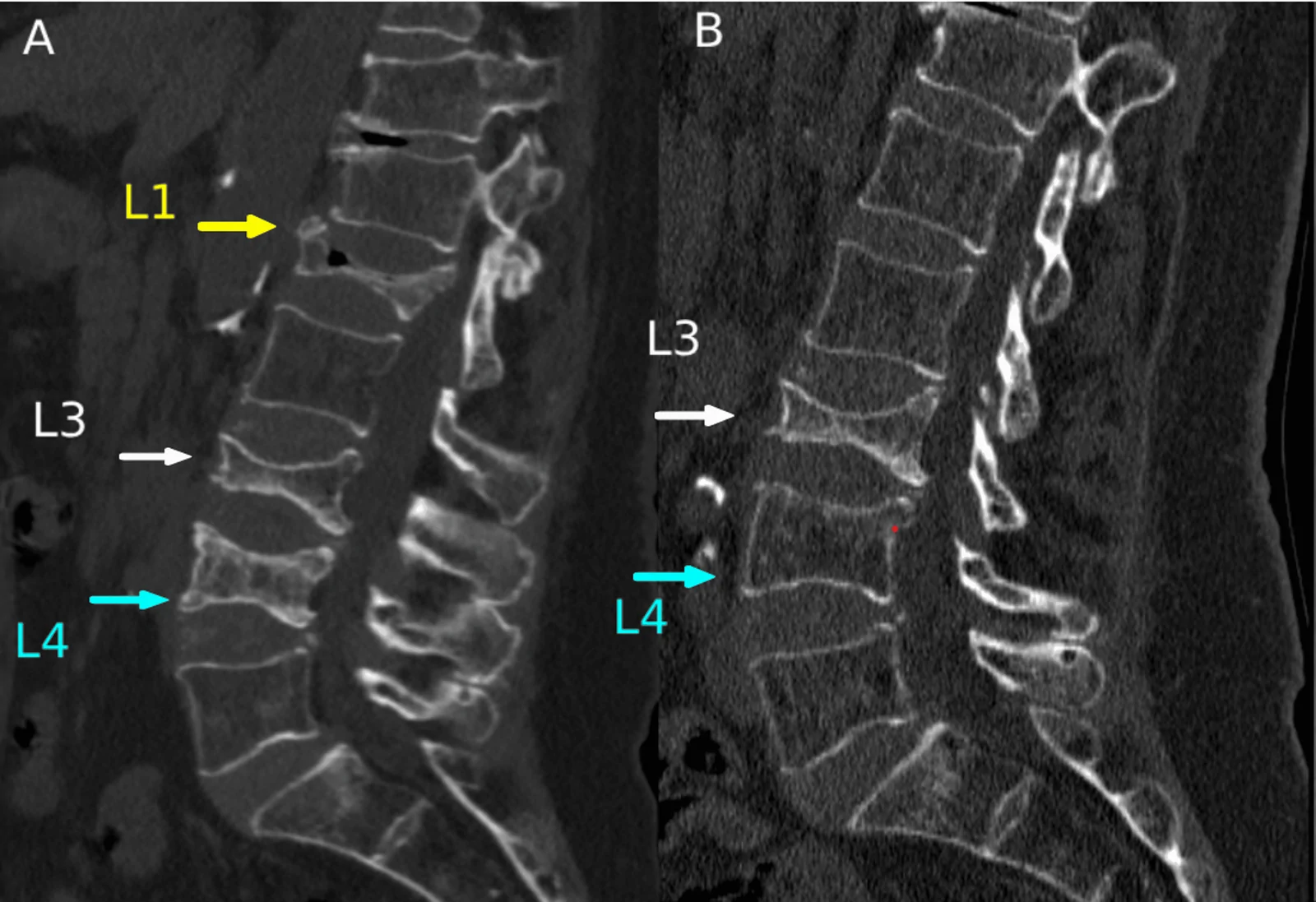
Lumbar spine CT scans (A) at presentation and (B) two months earlier. (A) L1 collapse with canal retropulsion and vacuum phenomenon (yellow arrow), L3 refracture deformation (white arrow), and progression of L4 fracture (bright blue arrow). (B) L4 fresh biconcave swelled-front type fracture, involving the anterior and posterior wall, and deformation of L3 refracture.

At presentation, full blood count was normal, IgG 1.93 g/dL, β2-microglobulin 3.66 mg/L, and κ FLC 62.3 mg/L. The FLC ratio was elevated at 5.11 compared to prior measurements over the preceding two years (1.91 and 3.69), indicating an increase in disease burden. The bone profile is shown in Table 1. Serum PINP levels were comparable to the discontinuation arm in the off-treatment extension of the primary phase III DMAb efficacy study (50 μg/L, at nine months after the last DMAb injection) [13]. Serum NTx levels were higher than the median premenopausal normal value (12.5 nBCE), consistent with the rebound phenomenon (~10 weeks delay), recent fractures, and renal failure. Overall, the findings indicated a low-risk SMM with possible evolving change and/or rebound bone resorption.

**Table 1:** Bone profile at presentation and follow-up. ^a^Normal premenopausal reference range Resuming denosumab suppressed serum NTx close to the lower premenopausal value at three months, with early uncoupling of bone formation. PINP was elevated at three and seven months and decreased to <35 ng/mL at 18 months. Parathyroid hormone elevation at three months reflected the relative hypocalcemia that is caused by suppression of osteoclast activity.

Parameters	Presentation	3 months	7 months	18 months	4 years	Reference range
Months since last DMAb injection	8.5	3	4	6	4	-
Creatinine	1.17	1.13	1.05	1.25	1.12	0.6-1.2 mg/dL
Albumin	37.6	34	36	35	34	32-50 mg/dL
Calcium	9.8	9.2	9.1	9.1	10.2	8.3-10.6 mg/dL
Phosphate	3.9	3.7	4	3.8	3.8	2.4-5.1 mg/dL
Alkaline phosphatase	51	44	34	25	27.7	27-130 U/L
Parathyroid hormone	29.1	54.6	27.4	28.7	14.8	10-95 pg/mL
Vitamin D, 25-hydroxy	43.1	41.3	42.9	24.37	32.2	<25 ng/mL
Collagen I propeptide (PINP)	40.4	53.8	50.8	32.5	17.7	15-60 ng/mL^a^
Collagen N-telopeptide (NTX)	13.5	7.5	8.4	8.8	5.5	6.2-19 nmBCE^a^

Bone quality was very poor. Review of the available bone density scans demonstrated a 14% femoral bone loss over the preceding two years (0.680 versus 0.791 g/cm²), despite DMAb therapy. Hounsfield units (HU) in L1 prior to collapse (Figure 4) and L2 was a contraindication for instrumentation (43.44 and 31.97, respectively). 

**Figure 4:**
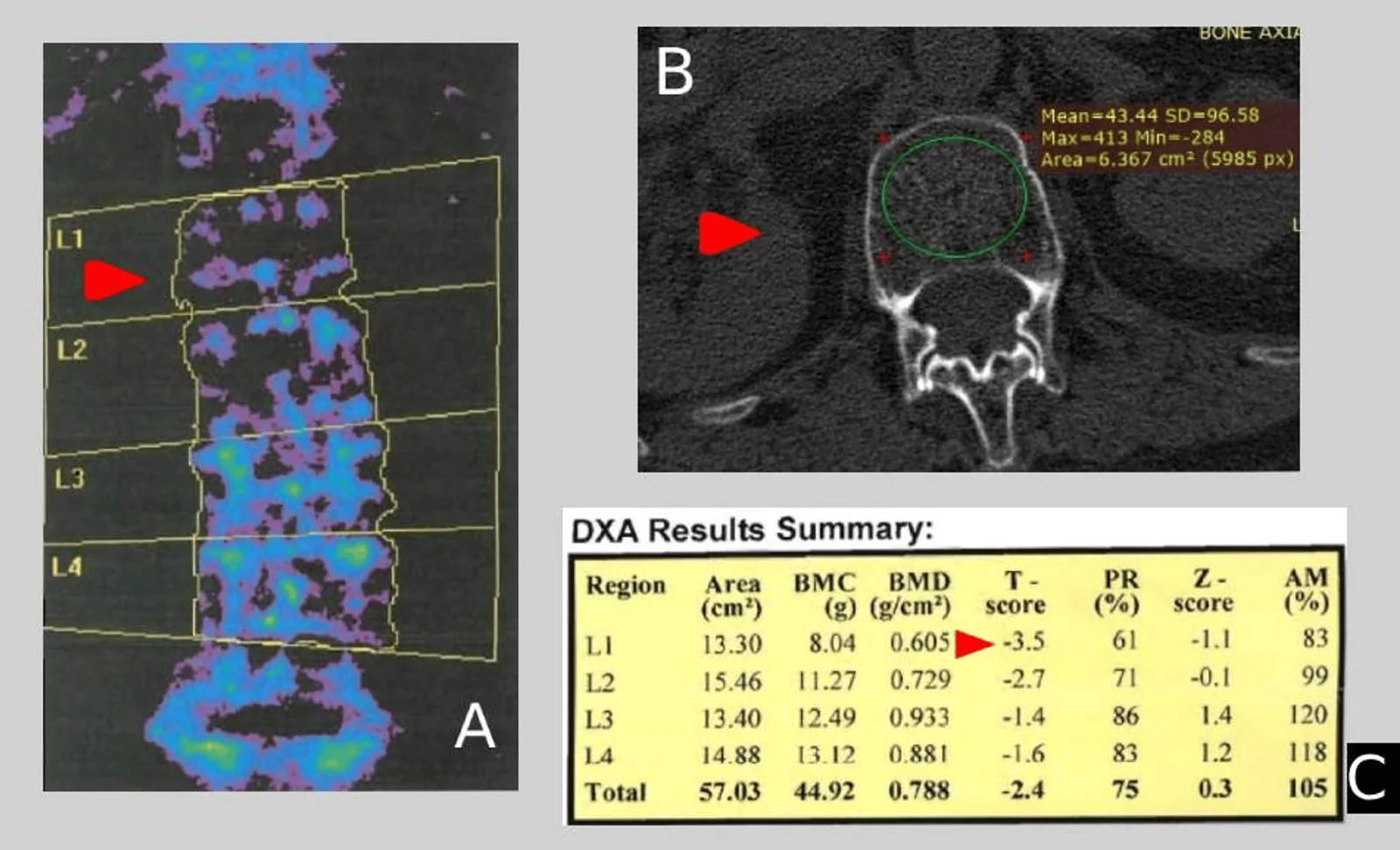
Bone quality evaluation. L1 vertebra (red arrow) showed severe osteoporosis (T-score= -3.5 SD) in (A,C) dual-energy X-ray absorptiometry (DXA) and (B) Hounsfield unit measurements on CT scan, prior to collapse. (A,C) False osteopenia at L3 and L4 reflects fracture changes (L3) and scoliosis (L4).

Administering DMAb at six months plus approximately seven weeks did not prevent the L3 refracture earlier. However, DMAb was resumed rather than transitioning to ZOL, given the 2.5-month delay since the last dose and the very high imminent fracture risk, which made a late switch to ZOL more risky. Percutaneous balloon kyphoplasty was performed at L1, L3, and L4 levels to reduce pain and improve mobilization. Intraoperative biopsies were obtained from L1, L2, and the posterior iliac crest. Cement was prophylactically injected into L2 and L5 using the balloon technique; delivery at L5 was impeded by high bone resistance and halted when leakage was observed. Biopsies were negative for myeloma involvement. Histology showed osteonecrosis at L1, traditionally associated with reduced bone formation. L2 biopsy demonstrated age-appropriate marrow cellularity with all three hematopoietic lineages, 3% CD138⁺ plasma cells with κ and λ light chains. Iliac marrow showed preserved trilineage hematopoiesis, with 1% CD34⁺ granulocytic precursors and scattered B and T lymphocytes. In the absence of whole-body MRI, the decision regarding systemic therapy was open to clinical ambiguity. However, IgG and FLC ratio values fell below 20/2/20 Mayo thresholds. Lenalidomide was not locally licensed, and the alternative to surveillance would be enrollment in a clinical trial involving dexamethasone, with risk of more fractures. The limitation of WB LDCT to detect lesions <5 mm, and the risks of enrollment in a clinical trial were discussed with the patient. Continuing surveillance was considered reasonably safe. 

Pain was immediately relieved and remained well controlled. At four weeks, the patient could walk independently indoors and perform activities of daily living. At three months, she had resumed community socialization, although she reported intermittent low back pain. Serum NTX was suppressed by 44% versus presentation (Table 1). The timing of the fractures relative to DMAb delays indicated suboptimal effectiveness at the end of six months. DMAb 60 mg administered every four months for two doses maintained serum N-terminal telopeptide (S-NTx) within the lower 30% of the premenopausal reference range before the next injection (Table 1). A new superior end-plate compression occurred in L2 (Figure 5).

**Figure 5:**
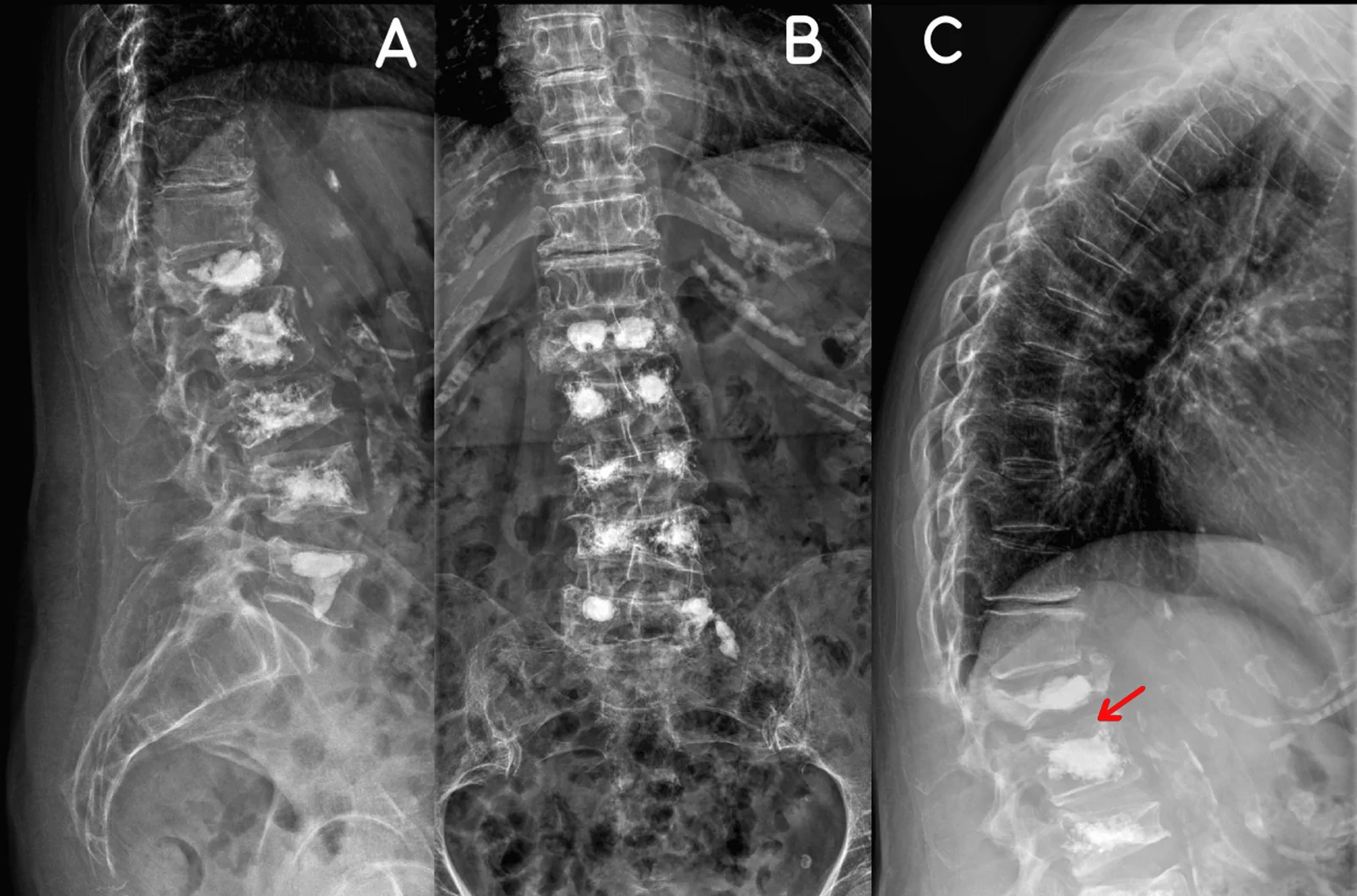
X-rays at six months post-kyphoplasty. (A) Lateral and (B) anteroposterior lumbar spine and lateral thoracic spine (C) X-rays; evidence of post-kyphoplasty L2 superior end-plate compression fracture (red arrow).

At the nine-month follow-up, BMPCs were 10%. A new WB LDCT ruled out osteolytic lesions but demonstrated progression of canal stenosis at L1 (Figure 6). The patient had no neurologic symptoms and no upper motor neuron or long-tract signs. Deep anal pressure and voluntary contraction were normal. After completing one year, the patient was switched to a six-month DMAb regimen.

**Figure 6:**
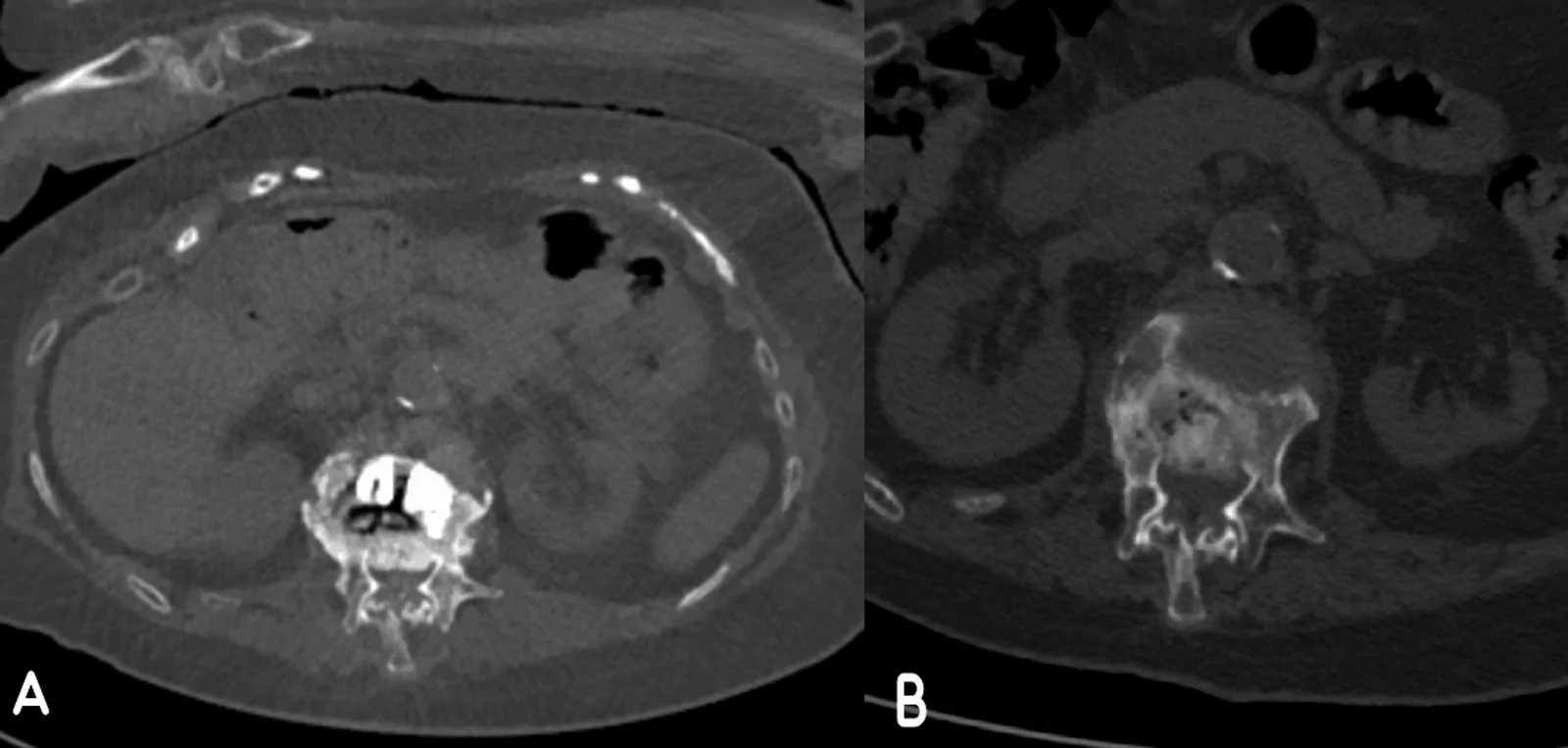
Progression of spinal canal stenosis at L1. (A) Whole-body low-dose CT (WB LDCT) at nine-month follow-up compared to (B) L1 canal retropulsion on lumbar spine CT at presentation (axial sections).

At four years, thoracic kyphosis had gradually progressed (Figure 7). IgG was 2.21 g/dL, κ FLC 71.6 mg/L, FLC ratio 3.81, and β2-microglobulin 4.29 mg/L. There were no new vertebral fractures, neurologic symptoms, or DMAb-related complications.

**Figure 7:**
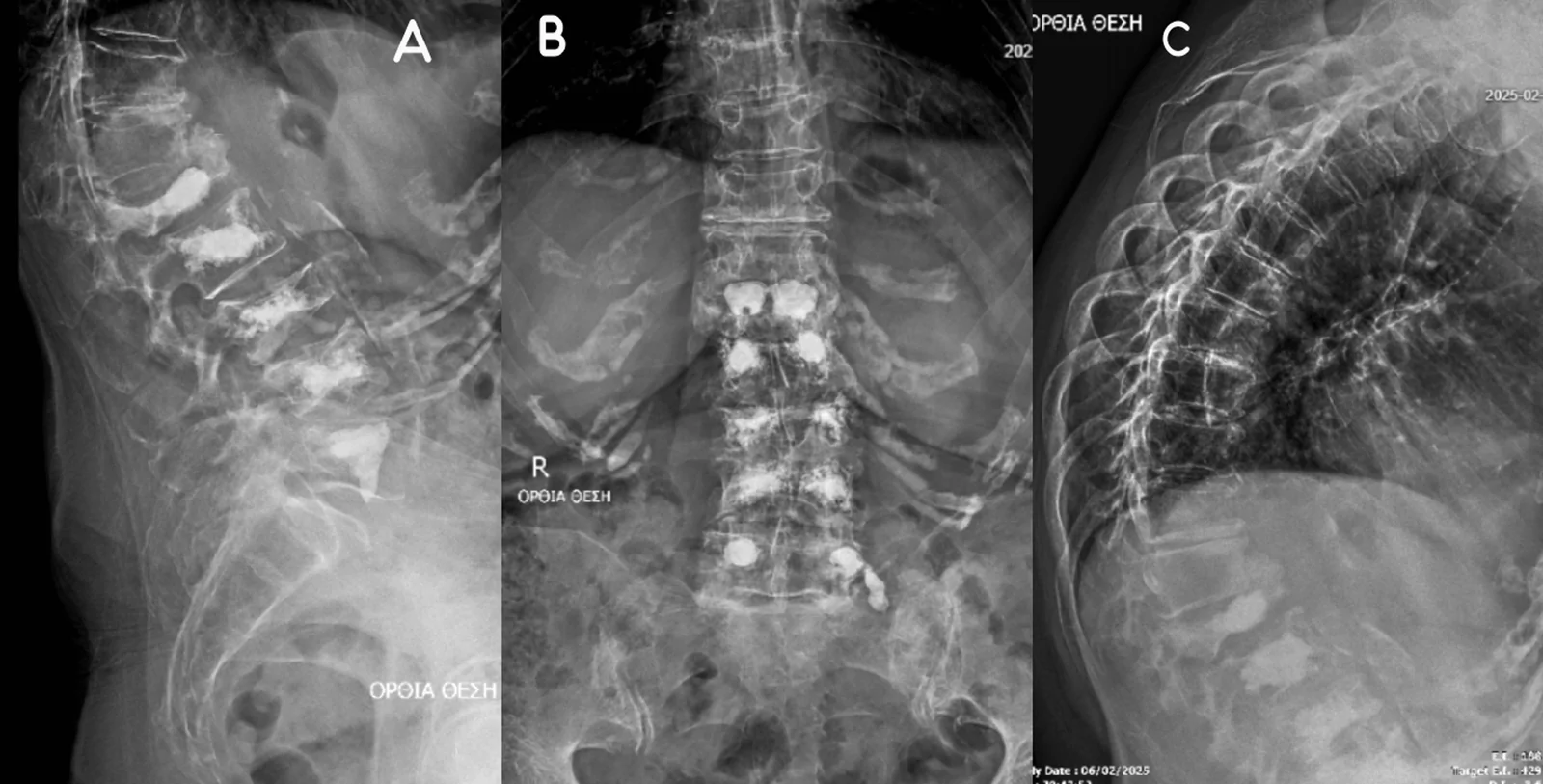
Standing lumbar and thoracic spine X-rays at four-year follow-up. (A,B) Lumbar spine and (C) thoracic spine with hyperkyphosis.

## Discussion

Evidence-based strategies for osteoporosis in patients with MGUS/SMM remain limited. RCTs support alendronate or ZOL 4 mg IV every six months in MGUS, while consensus supports ZOL every six to 12 months in SMM not requiring systemic therapy. Our patient's MGUS-related osteoporosis therapy was escalated to DMAb 60 mg every six months. The option favored the patient's CKD stage 3b. However, that choice carried major drawbacks: lack of data on effectiveness in this setting, need for strict adherence, and no defined exit strategy. These concerns proved clinically significant. The patient shifted to low-risk SMM and subsequently developed three vertebral events. Diagnosis was complicated by preceding delays in DMAb dosing. A limitation of this case was the lack of MRI, which is central to excluding focal marrow lesions. Surveillance was chosen despite the gradual biologic progression of the underlying plasma disorder, because the Mayo stratification indicated a low- to intermediate-risk SMM, which was confirmed as low risk by the marrow biopsy at nine-month follow-up. 

At presentation, the imminent fracture risk for our patient was very high, and DMAb injection was already 2.5 months delayed. The intensity of the rebound phenomenon and the associated fracture risk increase over time from DMAb onset [13]. Exiting DMAb after long-term therapy (four years in our case) and transitioning to ZOL every six months [14] or tapering DMAb while administering ZOL was considered risky for more rebound fractures. Unlike DMAb, bisphosphonates have a weak effect on osteoclast precursors and newly differentiated osteoclasts, which accumulate during the early rebound period [14]. Additionally, an RCT comparing the effect of timing of commencing ZOL after DMAb, nine versus six months after the last injection, showed that new fractures occurred exclusively in the nine-month group [15]. 

Resuming DMAb to reverse the rebound phenomenon is a common successful strategy. Unlike ZOL, which requires an increase in dosing in very high-risk patients [14], resuming DMAb follows the regular scheme. Interestingly, administering DMAb 60 mg at 6.9 weeks of delay did not prevent L3 refracture prior to presentation. Thus, we decided to shorten the time between DMAb injections and monitor with BTMs. Our approach was based on findings of the phase II varying dose study; DMAb 30 mg every three months effectively suppressed carboxytelopeptide collagen I (CTx), while the duration of the suppression was dose-dependent [16].

Serum BTM interpretation relied strongly on the time-dependent action of DMAb. BTMs might be elevated in high-risk MGUS/SMM, but findings are conflicting, and BTMs cannot be used for disease progression follow-up. There is no consensus on target BTM levels for monitoring DMAb response. Given DMAb's greater potency, target BTM levels are expected to be lower than the respective bisphosphonate targets (CTX <280 ng/L, P1NP <35 ng/mL). In our case, administering 60 mg of DMAb at three months and then every four months for one year maintained S-NTx before the next injection of DMAb within the lower 30% of the premenopausal range. This was expected to be effective since, in the FREEDOM bone turnover markers substudy, only 60% of women had resorption marker values below the premenopausal reference level at three years [17]. However, PINP did not decrease below the mean premenopausal level (<35 ng/mL) in a timely manner, and a new fracture occurred at L2 within six months. Therefore, a higher DMAb dose regimen may be more suitable. Two small prospective studies exploring the effect and safety of DMAb 120 mg monthly (n = 12) [18] or romosozumab (n = 8) [19] might be helpful to design strategies for BMD improvement and microarchitecture preservation in patients with SMM. 

In our case, L1 was almost a vertebra plana that occurred acutely. Prevalent fractures in T11 and T12 contributed to the kyphotic angle. The presence of a vacuum cleft was a predictor of non-union and a pain generator. Instrumentation was high risk for complications, and the purpose of kyphoplasty at L1 was to create a cement union and prevent further collapse or even restore the anterior height. The technical difficulties and risks of cement dislodgement, displacement, and leakage were discussed with the patient. At follow-up, L1 protruded more into the spinal canal, without myelopathy symptoms. L3 was a 10-month refracture (Figures 2A-2B) on top of a seven-year-old single end-plate compression (Figure 2C), which progressed to OF4 within the following eight months (Figure 3B). The severity of osteoporosis and osteonecrosis at L1 were negative predictors for L3 healing. VERTOS IV study showed that 40% of OVFxs that are treated conservatively continued to have disabling pain at 12-month follow-up [20]. VERTOS V demonstrated benefit from vertebroplasty at OVFxs with >3 months chronicity that were still healing (bone edema on MRI) [21]. Thus, the choice of L3 augmentation was based on VERTOS studies, although there was no MRI available. L4 had a clear indication for cement augmentation: pain, progression, and impairment of independent ambulation [11,12]. L2 carried a very high imminent risk of fracture due to age, recent fractures, thoracolumbar location, altered biomechanics from adjacent L1 and L3 fractures, BMD T-score -2.7, HU<55 [22], and biopsy. Nevertheless, conservative management entailed the risk of a fracture at L2 that would further increase the local kyphotic angle and worsen the canal stenosis. Evidence for prophylactic versus therapeutic kyphoplasty in preventing new fractures derives mainly from meta-analyses including heterogeneous small studies [23] and case reports. The superior endplate fracture at L2 despite kyphoplasty (Figure 5), along with resistance during cementing at L5 in our patient, may support a selective empirical approach guided by very low HU values.

Discontinuation of DMAb was previously linked to avascular necrosis in a woman who presented with multiple vertebral osteonecroses after weaning off 10-year DMAb therapy. The mechanism was attributed to the absence of osteocytes, evidenced by empty lacunae in histomorphometry [24]. We postulate that L1 osteonecrosis in our patient was related to rebound resorption with uncoupled reduced bone formation. As shown by Antonacci et al., avascular necrosis is in any case closely intertwined with osteoporosis. In a cadaveric study, they consistently identified avascular necrotic areas with unreactive marrow in osteoporotic fractured vertebrae, as well as in most adjacent vertebrae harboring radiographically occult endplate and cortical fractures [25]. Romosozumab is characterized by a high potency in promoting cancellous bone healing, and reduction of pain in patients with OVFxs treated conservatively with romosozumab was superior to vertebroplasty plus DMAb at 12 months [26]. 

Cementing multiple vertebral fractures in a single session is considered a safe and effective treatment option (grade C) [11], especially if limited to five levels [27], and up to 30 cc cement total volume. The DGOU/AO recommended surgery with instrumentation for the majority of OF4 fractures [12]. However, in a validation study, about 45% of the OF4 fractures were treated by vertebral augmentation and only 9% with open surgery [28]. Vertebral augmentation represents a realistic option for patients without neurological deficit when the primary goals are pain relief and restoration of daily functioning, although it may be insufficient in preventing the progression of long-term hyperkyphosis. As anticipated in our case, kyphoplasty did not prevent the progression of kyphosis over the four-year follow-up period (Figure 7). 

Overall, resuming DMAb with shortening the interval between doses for the year following the fractures, combined with balloon kyphoplasty, reduced pain, improved mobilization, and stabilized the fracture disease. 

## Conclusions

A patient with MGUS or low-risk SMM may exhibit poor response to therapies for postmenopausal osteoporosis. DMAb use in patients with osteoporosis and MGUS/SMM may be complicated by the overlap of DMAb time-dependent action and evolving bone marrow disease.

In our case, resuming DMAb 60 mg at seven- and 10-week delays did not prevent fractures. Nevertheless, shortening the time between doses attenuated the rebound bone resorption but did not suppress it below the lower premenopausal reference levels. Ultimately, the combination of monitored antiresorptive therapy and balloon kyphoplasty represents a therapeutic option for patients with MGUS/SMM related osteoporosis and very high imminent fracture risk, when there is an indication for surgery without neurological deficit.
